# A “Hole Punched Plate” method for easy generation and harvesting of microconidia in the dermatophyte *Trichophyton rubrum*

**DOI:** 10.1016/j.heliyon.2018.e00676

**Published:** 2018-07-05

**Authors:** Wolfram Siede

**Affiliations:** Santa Fe BioLabs LLC, Fort Worth, TX, USA

**Keywords:** Biotechnology, Cell biology, Microbiology

## Abstract

Handling of the medically important dermatophyte *Trichophyton rubrum* in the laboratory typically requires the generation of spores — for storage, treatment and plating when needed. The described method allows technically simple but efficient generation and harvesting of microconidia by cutting holes in Sabouraud dextrose agar medium that is covered by a mature *T. rubrum* mycelium.

## 1 Introduction

The dermatophyte *Trichophyton rubrum* and related species (such as *T. tonsurans*, *T. mentagrophytes*) are of significant medical interest since they are the source of common disorders of skin and hair [1, 2]. For example, they may give rise to athlete's foot, ringworm and fungal infection of nails (tinea pedis, tinea corporis, tinea unguium).

Routine handling of these molds in the research laboratory typically requires the efficient generation of spores (micro- and/or macroconidia, the latter break into arthroconidia), so that they can be harvested, stored, treated and plated when needed. It is often time-consuming and not straightforward to generate a homogenous spore suspension of sufficient density from cultured *T. rubrum* and its various patient isolates. Certain methods have been established for generation of microconidia and arthroconidia in *T rubrum* [3, 4, 5, 6, 7, 8]. For example, a method for large scale production of *T. rubrum* microspores that employs high CO_2_ tension has recently been introduced [9]. The method described here, however, is based on cutting holes into standard medium agar plates where a mature mycelium is grown. In terms of ease, required time and spore yield, we found this method to be superior to others.

## 2 Material and methods

**Fig. 1:**
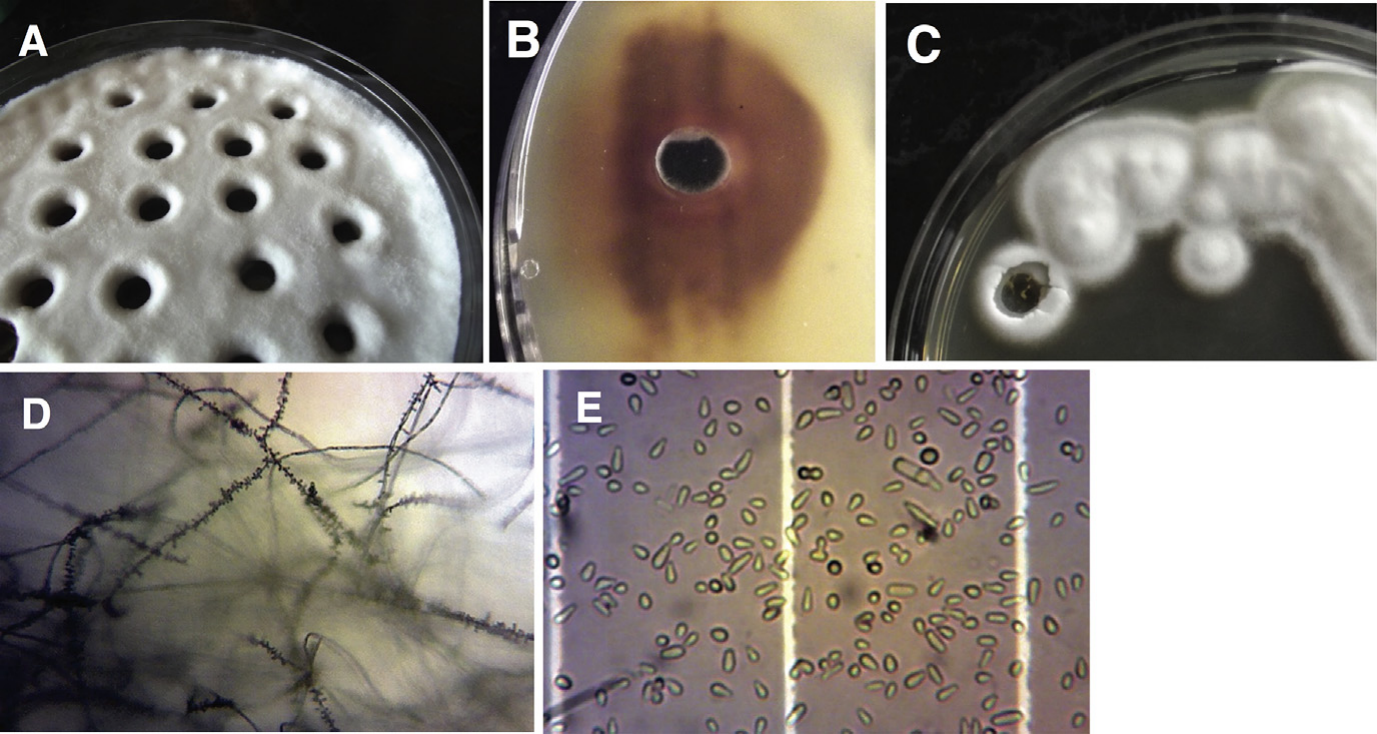
Holes cut into agar plates covered by a mature mycelium stimulates microconidia formation in *Trichophyton rubrum*. After 8 days of incubation at 30 °C, holes are cut into the mycelium and agar plugs are removed. After about 2 days of further incubation, regrowth is often apparent around the periphery of the openings (A). At the same time, red pigmentation develops surrounding the holes and is clearly visible if the plate is viewed from the bottom (B). The same procedure may also be performed on well-separated single clones (C). Microscopic examination reveals the development of microconidia in the area around the holes, as indicated by the typical “birds on a wire” appearance of the mycelium (D); subsequently, microconidia can easily be collected as a suspension (E).

In this agar slicing method, we use a Sabouraud dextrose medium composed of 1.6–2% agar, 1% meat peptone and 4% dextrose, the latter added after autoclaving from a 20% stock. The pH of the medium stays unadjusted and is typically around pH 7. Antibiotics may be added to suppress any bacterial growth but adjusting the pH to the acidic range (∼5.6) is not recommended since it will result in lower spore yields. To start, an existing microconidia sample (or any source of spores/cells) is used for plating or streaking at a density that leads to a dense mycelium cover of a petri plate within a few days. After two days incubation at 30 °C, plates are typically sealed with Parafilm to prevent excessive drying and as a means to avoid spreading of spores of this BSL2 organism. After about 8 days of incubation in total (in the dark), holes of ∼6 mm diameter are cut into the now mycelium-covered agar, preferably by using a flamed cork borer or a similar cutting tool (Fig. 1A–C). The circular agar pieces are removed and discarded. The resulting holes should be not placed less than 10 mm apart. After continued incubation, additional growth may become evident around the holes (Fig. 1A). In roughly the same area, developing red pigmentation should be visible when the plate is viewed from the bottom (Fig. 1B). This appears to accompany formation of spores which are mostly microconidia in our case (Fig. 1D). In another *Trichphyton* species, carotenoid production has been associated with arthrospore formation [10].

After at least an additional 2 days of incubation after holes have been cut, the conidia can easily be harvested as follows. Sterile water or a buffer of choice is pipetted carefully onto zones of 3–5 mm surrounding the holes. After having let it stand for about 15 min to improve wetting, a sterile tool (such as flamed glass or plastic spreader, rubber policeman, cotton swab) is used to gently scrape the wet areas repeatedly. Care should be taken not to visibly scrape off mycelium. The fluid is guided towards the holes where it will accumulate and can easily be removed by pipetting. The remainder can be removed by tilting the plate. The procedure is repeated at least once. The combined fluid volumes may be centrifuged, the spore pellet may then be washed and resuspended at the desired density in the storage buffer of choice (e.g. suitable for freezing). Microconidia suspensions generated by this method are quite uniform, with less than 1% contamination by mycelium fragments (Fig. 1E).

For large-scale spore preparation, an entire petri dish can be covered with equally spaced holes (Fig. 1A). However, this method can also be applied on a diluting streak to harvest spore progeny from a single, well-separated mold clone (Fig. 1C). In this case, incubation periods may have to be extended slightly.

## 3 Results and discussion

The method described here is based on cutting holes into a mature mycelium of *Trichophyton rubrum* grown on standard Sabouraud dextrose medium and yields high spore numbers in as little as 10 days (Fig. 1 A–E). In terms of technical simplicity (e.g. no need for high CO_2_ tension), required time and spore yield (∼1 × 10^7^/ml of harvested suspension), we found it to be superior to other methods, such as extended growth on potato dextrose agar (yielding ∼ 2 × 10^6^ condidia/ml at ambient CO_2_ concentration).

We are not entirely sure about the mechanism behind this serendipitously found effect. Factors that stimulate conidiation and may play a role here have been more thoroughly studied in other filamentous fungi such as *Aspergillus* or *Neurospora* than in *T. rubrum* [11]. Interestingly, mechanical injury of mycelia has previously been reported as a trigger for conidiation in *Trichoderma atroviride* [12]. However, in our hands, just cutting into the mycelium was much less effective than removing the cut pieces of agar (data not shown). We suspect a combination of several factors. Aerial exposure of a mature mycelium grown submerged in the substrate together with mechanical injury and progressive drying around the edges of the holes may synergize to stimulate spore formation in our procedure.

It is of interest to explore if the method can be adopted for other molds that are otherwise difficult to sporulate. Our preliminary observations suggest that this might indeed be the case.

## Declarations

### Author contribution statement

Wolfram Siede: Conceived and designed the experiments; Performed the experiments; Analyzed and interpreted the data; Contributed reagents, materials, analysis tools or data; Wrote the paper.

### Funding statement

This research did not receive any specific grant from funding agencies in the public, commercial, or not-for-profit sectors.

### Competing interest statement

The authors declare no conflict of interest.

### Additional information

No additional information is available for this paper.
